# Supplementary material for: Antibody Generation and Immunogenicity Analysis of EBV gp42 N-Terminal Region
Source: Viruses. 2021 Nov 28;13(12):2380. doi: 10.3390/v13122380 (PMC8707153; doi:10.3390/v13122380)
Supplement: Supplementary file 1 [file viruses-13-02380-s001.zip › viruses-1430358-SI.pdf]

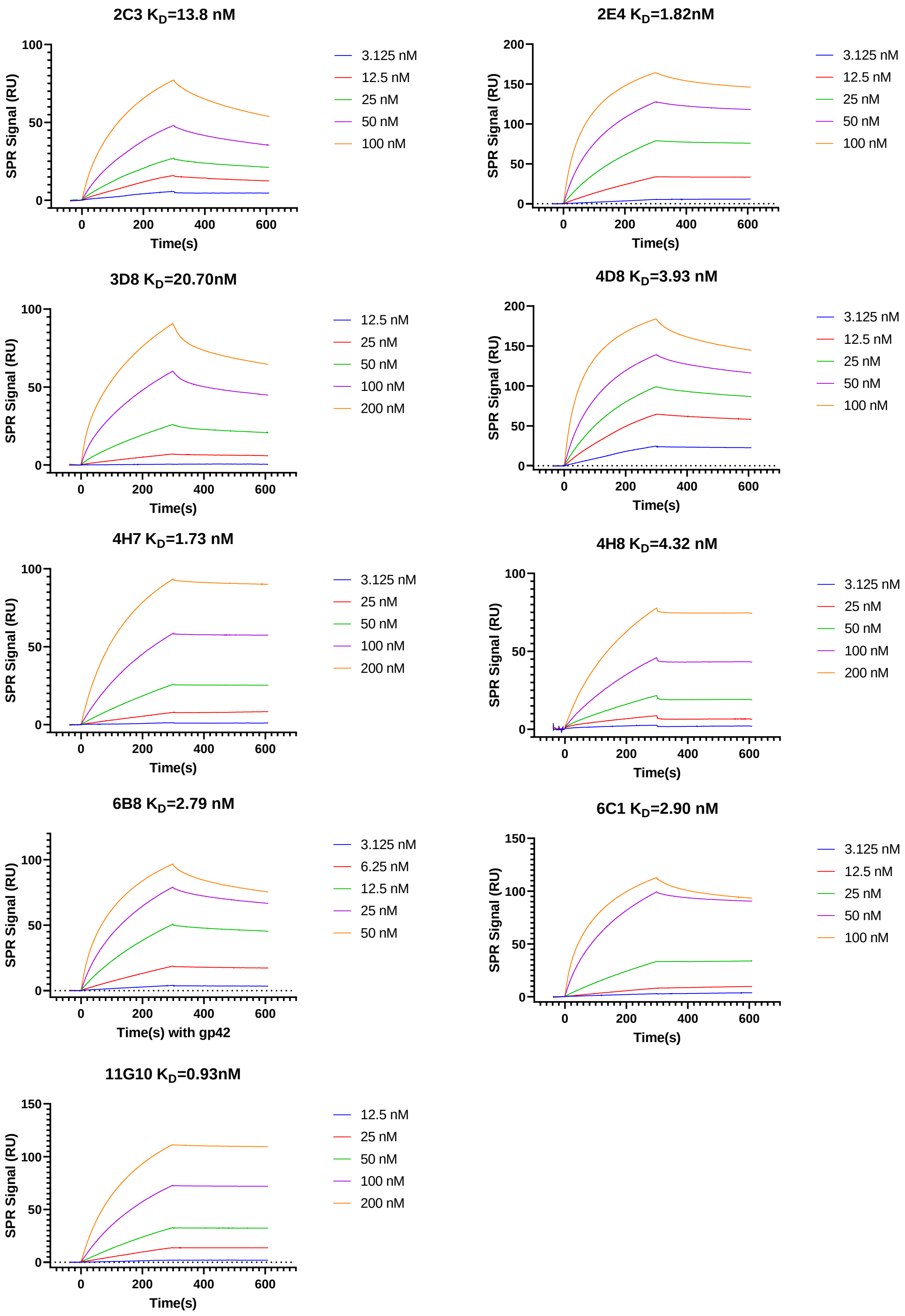

Figure S1. Binding affinity of 9 mAbs to gp42 conjugated to Sensor Chip CM5.

A

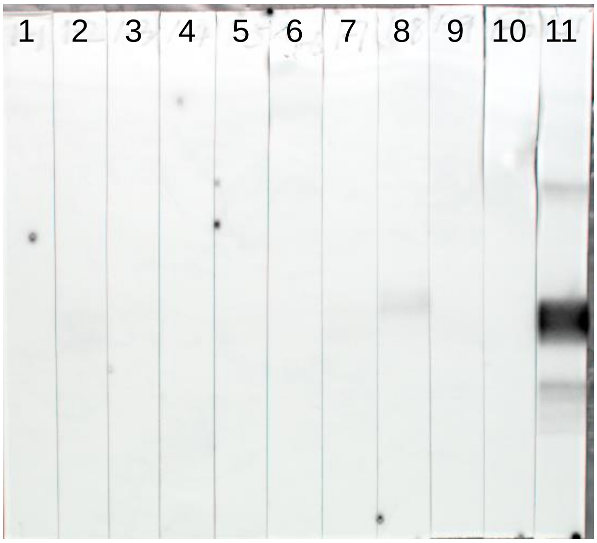

1. 2C3
2. 2E4
3. 3D3
4. 4D8
5. 4H7
6. 4H8
7. 6B8
8. 6C1
9. 11G10
10. 72A1
11. Anti-GST

B

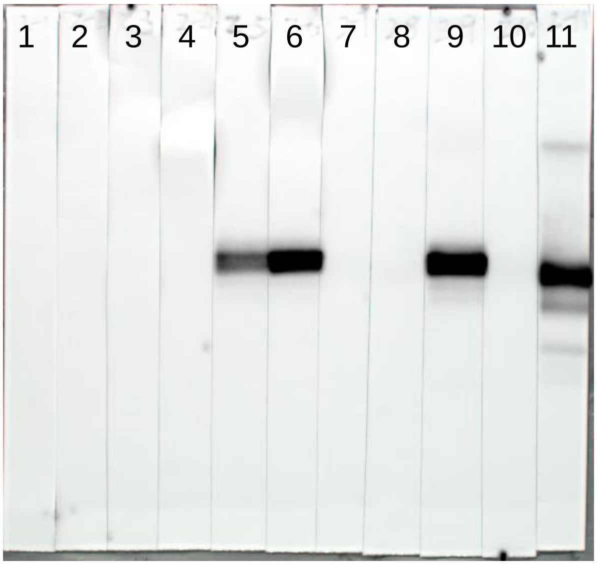

1. 2C3
2. 2E4
3. 3D3
4. 4D8
5. 4H7
6. 4H8
7. 6B8
8. 6C1
9. 11G10
10. 72A1
11. Anti-GST

C

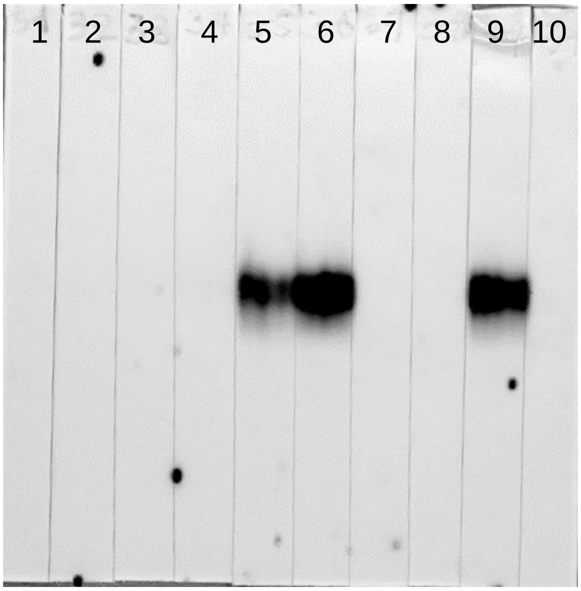

1. 2C3
2. 2E4
3. 3D3
4. 4D8
5. 4H7
6. 4H8
7. 6B8
8. 6C1
9. 11G10
10. 72A1

Figure S2. Western blot analysis of anti-gp42 mAbs against (A) gp42(residues 44-61), (B) gp42(residuese 67-81) and gp42 protein.

The mAbs used in western blot were listed on the right.

A

```
EBV-gp42:rhLCV-gp42
Identity= 79.64%(176/221)

1    MVSFKQVRVPLFTAIALVIVLLLAYFLPPRVRGGGRVAAAAITWVPKPNVEVWPVDPPPP
     ||||||||||||||:||||||||||:|||||| ||.|||||||||:.|.|||||.:||||
1    MVSFKQVRVPLFTALALVIVLLLAYYLPPRVR.GGQVAAAAITWVANPKVEVWPAEPPPP

61   VNFNKTAEQEYGDKEVKLPHWTPTLHTFQVPQNYTKANCTYCNTREYTFSYKGCCFYFTK
     ::||||||||||..|:.||||.||||||.||:||||:|||||||..||||:.  |||||.
60   IDFNKTAEQEYGKPEIDLPHWKPTLHTFKVPENYTKPNCTYCNTQKYTFSFSKRCFYFTP

121  KKHTWNGCFQACAELYPCTYFYGPTPDILPVVTRNLNAIESLWVGVYRVGEGNWTSLDGG
     .||.|:|||.||||||.||||||||::|||||| ||.| ||||||||:||||||||||||
120  QKHPWDGCFKACAELYECTYFYGPTANILPVVTGNLKASESLWVGVYKVGEGNWTSLDGG

181  TFKVYQIFGSHCTYVSKFSTVPVSHHECSFLKPCLCVSQRSNS
     .:.|||||||||||||| |.|||||||||| |||||||:|.:
180  NYQVYQIFGSHCTYVSKSSKVPVSHHECSFHKPCLCVSHRKD
```

B

```
EBV-gp42-44-81aa:rhLCV-gp42-43-80aa
Identity= 71.05%(27/38)

1  WVPKPNVEVWPVDPPPPVNFNKTAEQEYGDKEVKLPHW
   ||:.|.|||||.:||||::||||||||||..|:.||||
1  WVANPKVEVWPAEPPPPIDFNKTAEQEYGKPEIDLPHW
```

Figure S3. Alignment of EBV (Genbank ID: KF373730.1) and rhLCV (Genbank ID: AY037858) gp42 using DNAMAN.

(A) Full-length sequence and (B) N-terminal sequence of gp42 were analyzed. The identical amino acid was linked with vertical line.

A

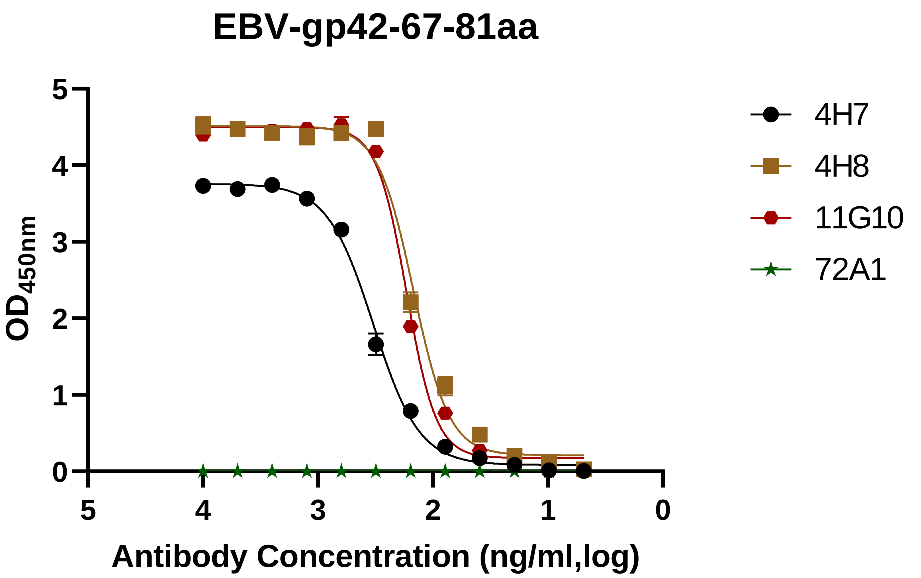

B

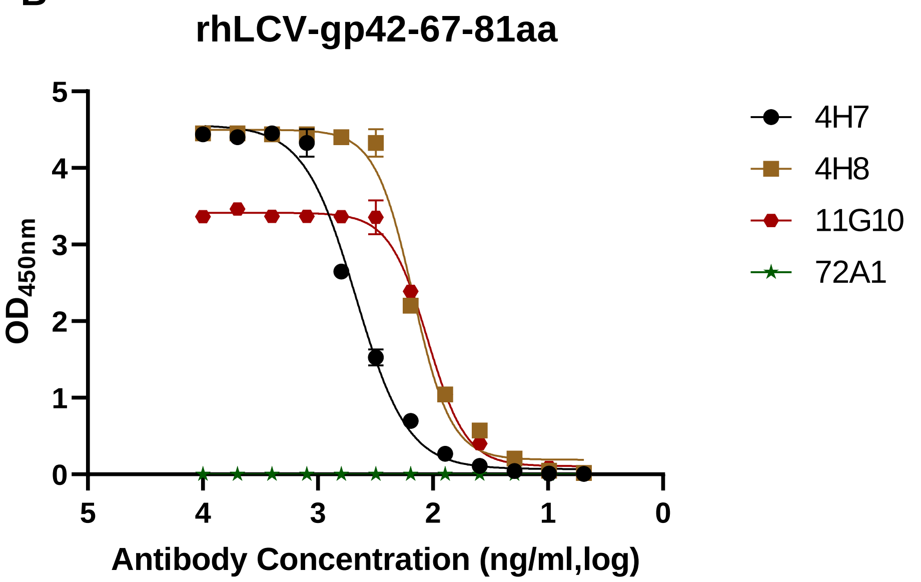

Figure S4. The reactivity of 4H7, 4H8 and 11G10 with synthetic peptides, (A) EBV-gp42(residues 67-81) and (B) rhLCV-gp42(residues 66-80).

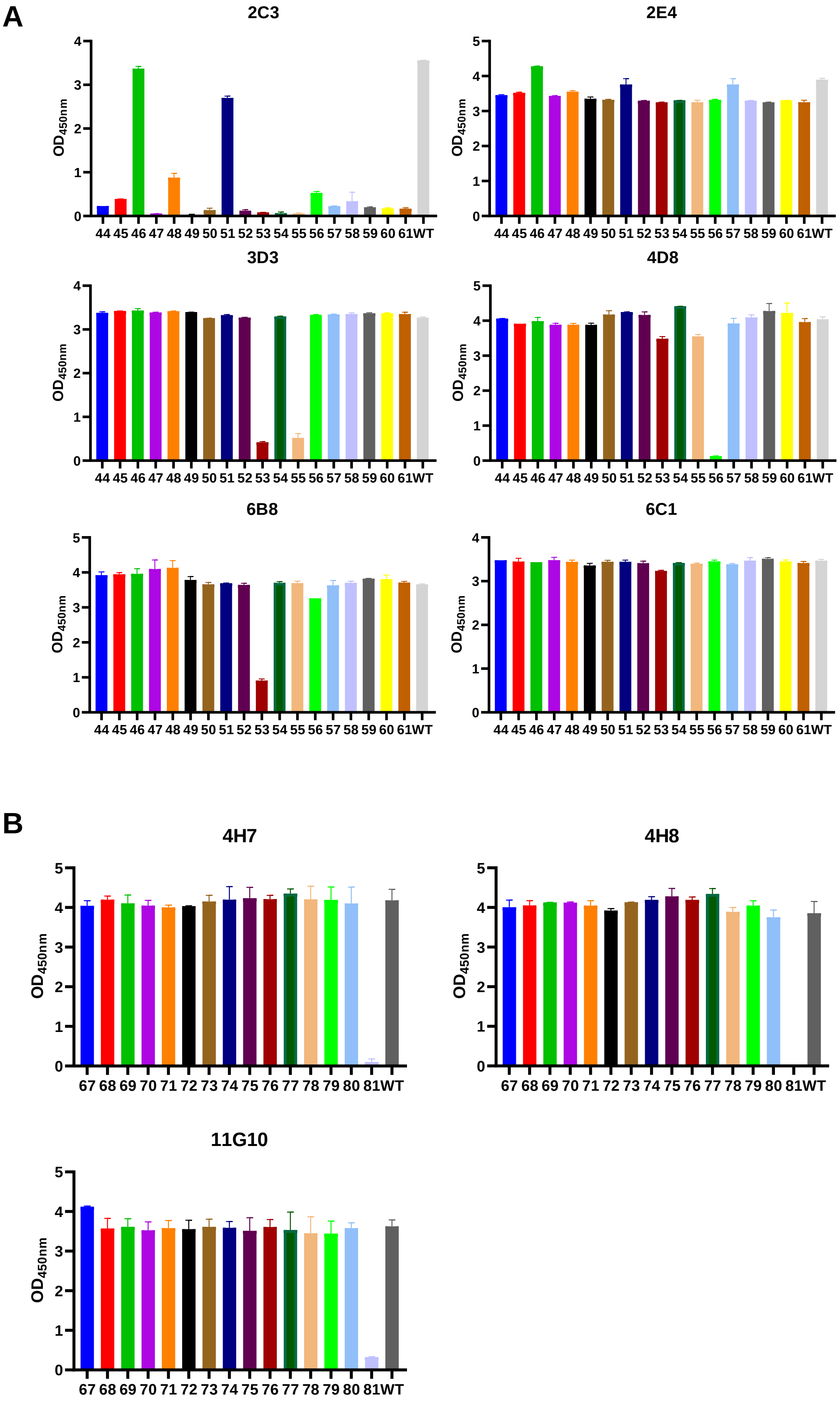

Figure S5. Alanine-scanning mutagenesis of gp42 N-terminal peptides. (A) gp42-44-61aa. (B) gp42-67-81aa.

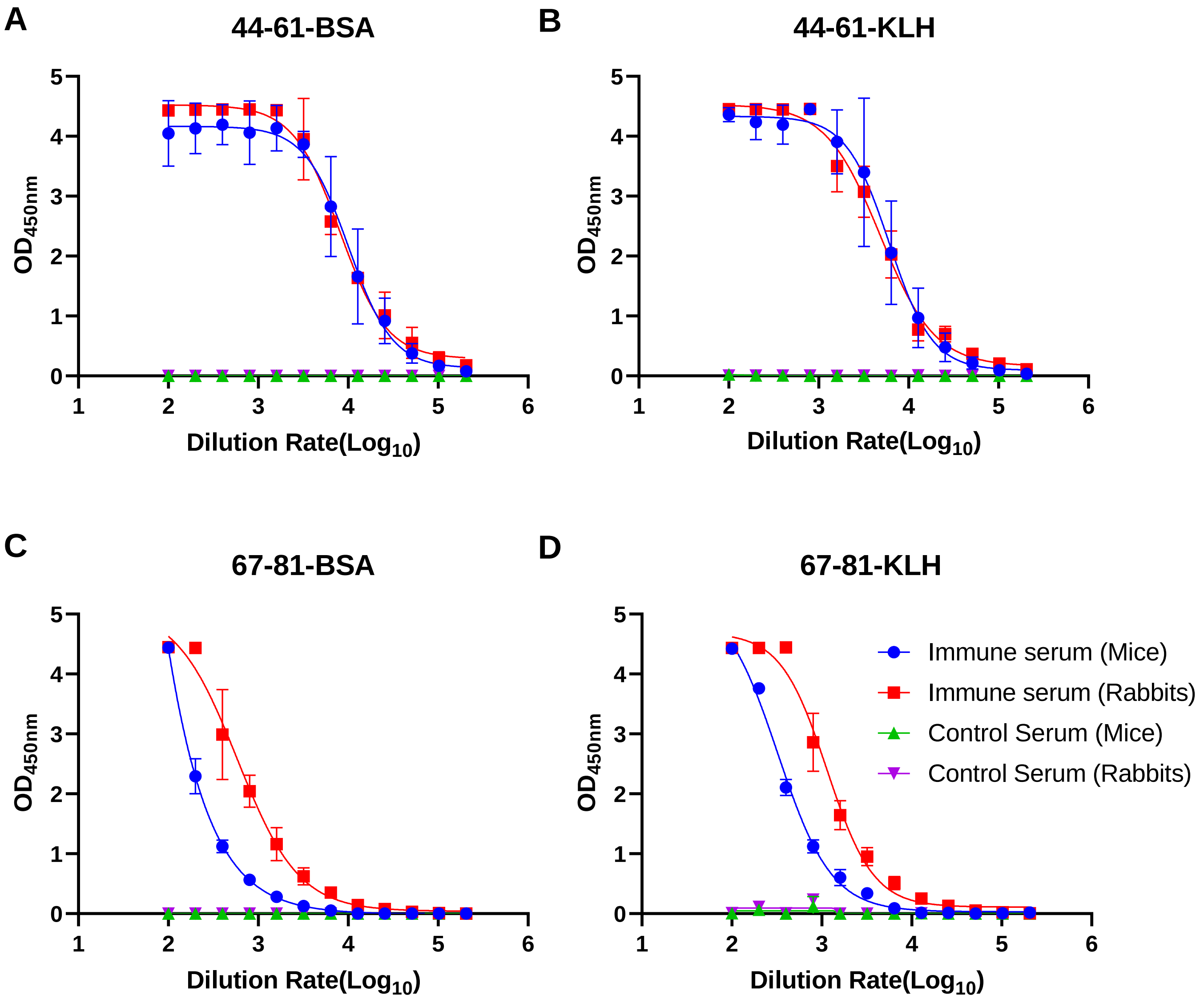

Figure S6. The reactivity of immune serum derived from mice immunized with gp42 protiens against BSA and KLH conjugated peptides, (A) and (B) gp42(residues 44-61), (C) and (D) gp42(residues 67-81).

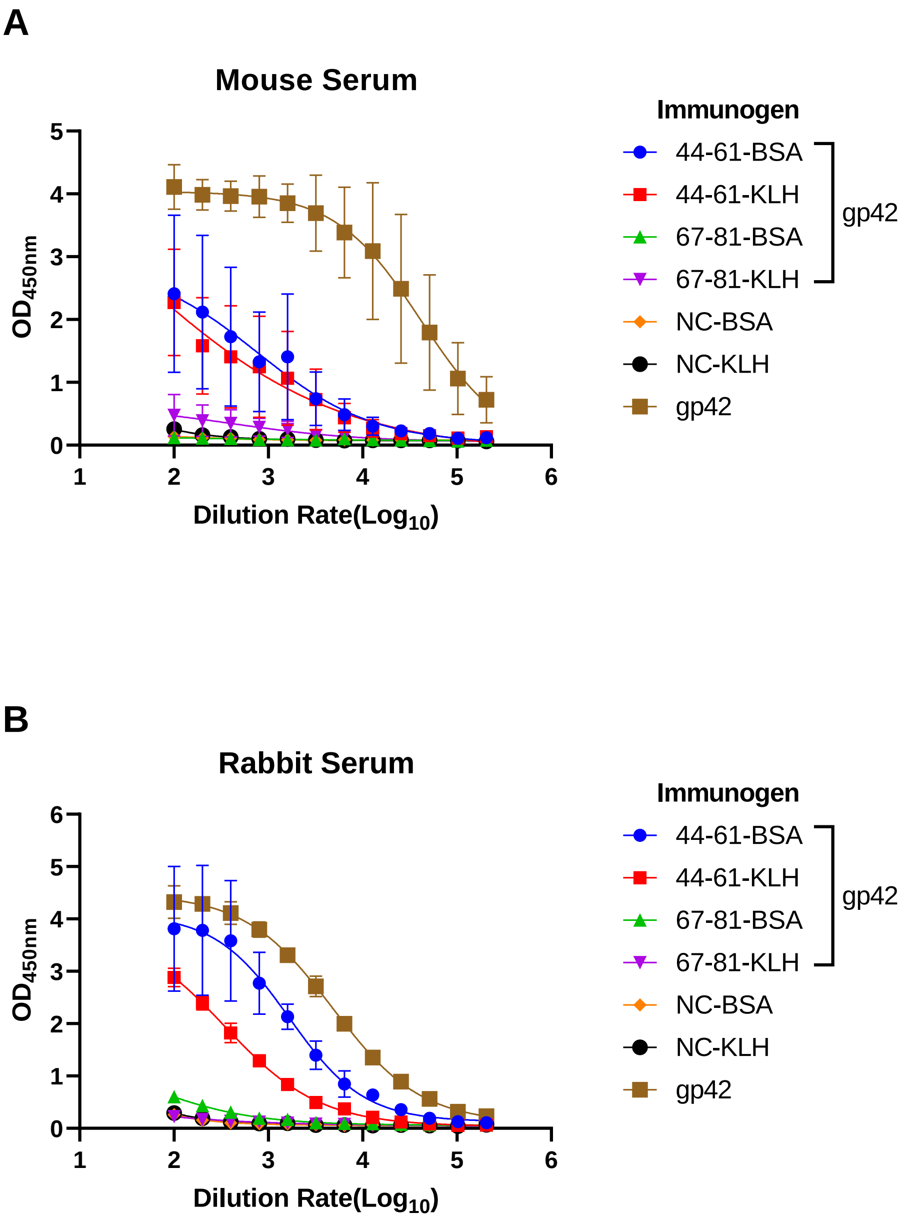

Figure S7. Immunized serum titers against gp42. (A) Mice and (B) rabbits immunized with KLH, BSA conjugated peptides.

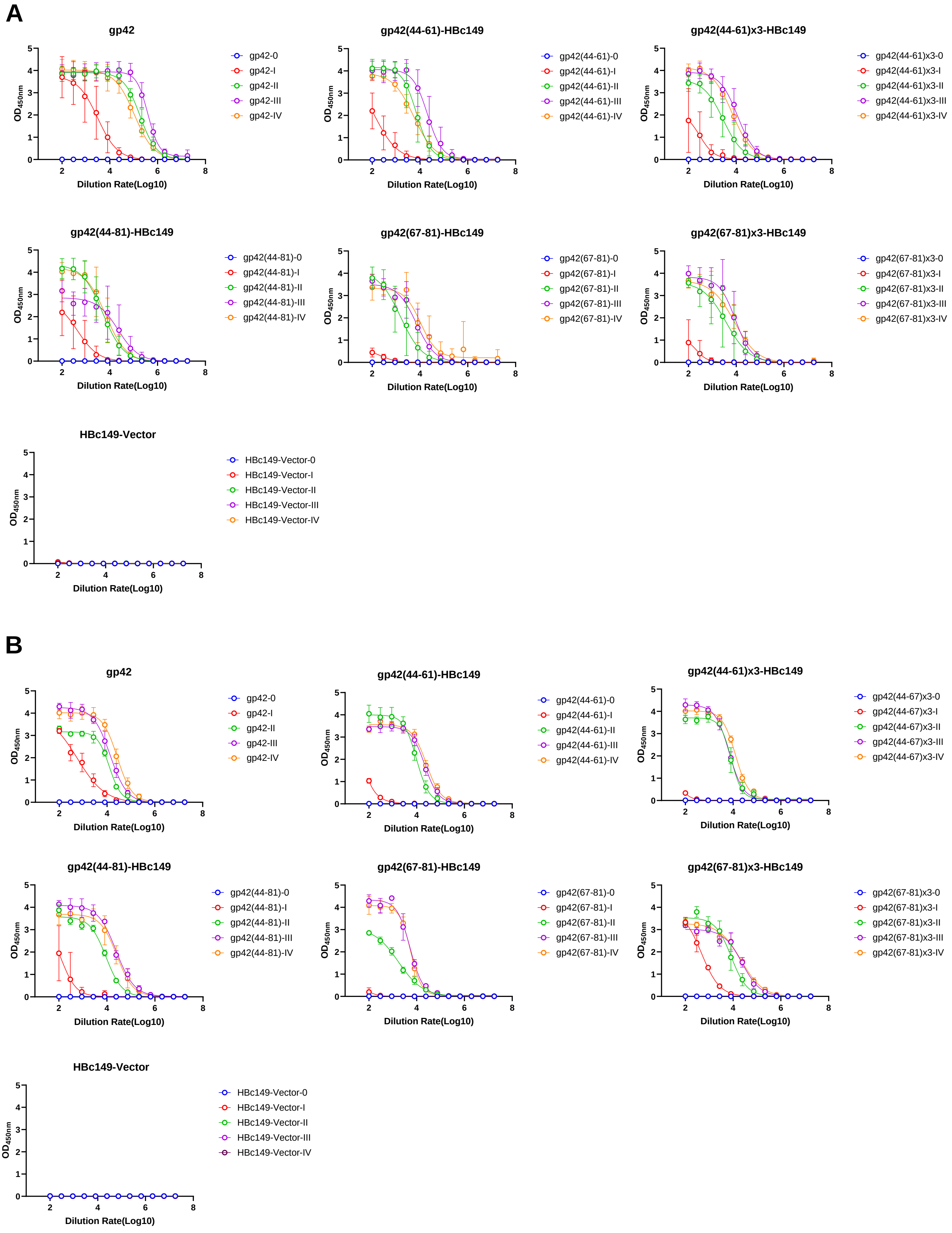

Figure S8. Antibody titers against gp42. (A) Mice and (B) rabbits immunized with chimeric VLPs carrying peptides derived from gp42 N-terminal region.

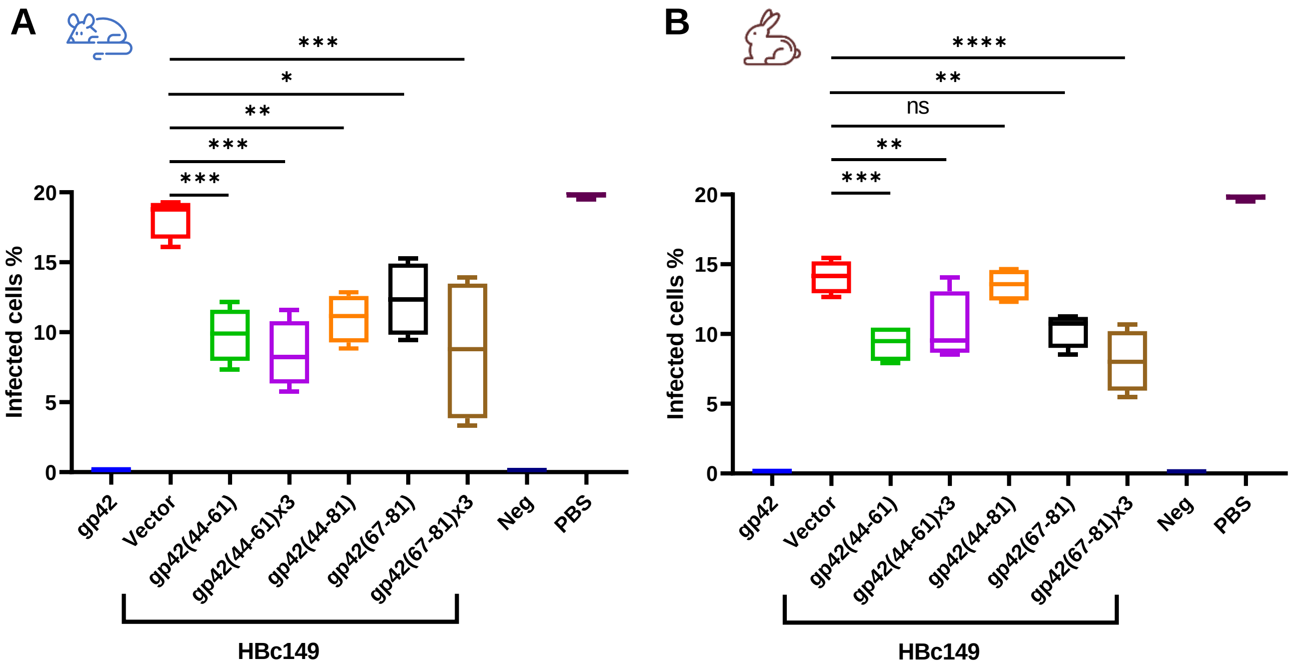

Figure S9. Serum samples collected at week 8 from (A) mice and (B) rabbits to block EBV infection into Akata cells. (*p≤0.05, **p≤0.01, ***p≤0.001)
